# Hybrid Silica Materials Applied for Fuchsine B Color Removal from Wastewaters

**DOI:** 10.3390/nano11040863

**Published:** 2021-03-28

**Authors:** Ion Fratilescu, Zoltán Dudás, Mihaela Birdeanu, Camelia Epuran, Diana Anghel, Ionela Fringu, Anca Lascu, Adél Len, Eugenia Fagadar-Cosma

**Affiliations:** 1 Institute of Chemistry “Coriolan Dragulescu”, Mihai Viteazu Ave. 24, 300223 Timisoara, Romania; ionfratilescu@acad-icht.tm.edu.ro (I.F.); ecamelia@acad-icht.tm.edu.ro (C.E.); danghel@acad-icht.tm.edu.ro (D.A.); mcreanga@acad-icht.tm.edu.ro (I.F.); alascu@acad-icht.tm.edu.ro (A.L.); 2 Neutron Spectroscopy Department, Centre for Energy Research, Konkoly-Thege Street 29-33, 1121 Budapest, Hungary; dudas.zoltan@ek-cer.hu (Z.D.); len.adel@ek-cer.hu (A.L.); 3 National Institute for Research and Development in Electrochemistry and Condensed Matter, P. Andronescu Street 1, 300224 Timisoara, Romania; mihaione2002@yahoo.com; 4 Civil Engineering Department, University of Pécs, Boszorkány Street 2, 7624 Pécs, Hungary

**Keywords:** PtNPs, porphyrin derivatives, hybrid silica materials, morphologic and textural characterization, AFM and SEM microscopy, SANS, fuchsine B, wastewater discoloration

## Abstract

Hybrid materials, with applications in fuchsine B color removal from wastewaters, were obtained by in situ incorporation of platinum nanoparticles and/or Pt-porphyrin derivatives into silica matrices. The inorganic silica matrices were synthesized by the sol-gel method, conducted in acid-base catalysis in two steps and further characterized by Nitrogen porosimetry, Small Angle Neutron Scattering (SANS), Scanning electron microscopy, Atomic force microscopy and UV-vis spectroscopy. All of the investigated silica hybrid materials were 100% efficient in removing fuchsine B if concentrations were lower than 1 × 10^−5^ M. For higher concentrations, the silica matrices containing platinum, either modified with Pt-metalloporphyrin or with platinum nanoparticles (PtNPs), are the most efficient materials for fuchsine B adsorption from wastewaters. It can be concluded that the presence of the platinum facilitates chemical interactions with the dye molecule through its amine functional groups. An excellent performance of 197.28 mg fuchsine B/g adsorbent material, in good agreement with the best values mentioned in literature, was achieved by PtNPs-silica material, capable of removing the dye from solutions of 5 × 10^−4^ M, even in still conditions.

## 1. Introduction

Known as a main source of industrial water, colored wastewaters require dye removal prior to any other treatment. Adsorption is considered to be an effective environmental technique for this purpose [1].

Mesoporous silica matrices (2–50 nm pore size) based on silicon dioxide nanoparticles are promising for wide applications both due to their low toxicity and high thermal stability and because of their facile preparation and surface functionalization enabling to tailor the porosity, the specific surface, the shape and size of the silica nanoparticles. Suitable combinations of these properties make silica nanoparticles ideal materials especially for specific medical [2] or environmental [3] adsorption and separation.

Among these, functionalized silica derivatives with metal nanoparticles having specific surface area over 500 m^2^/g are nowadays of wide interest due to their successful applications in detection [4,5], catalysis [6] and wastewater treatments [7]. The main advantage of these silica hybrid materials is their possibility to be reused due to easy separation from heterogeneous systems [8,9].

**Figure 1:**
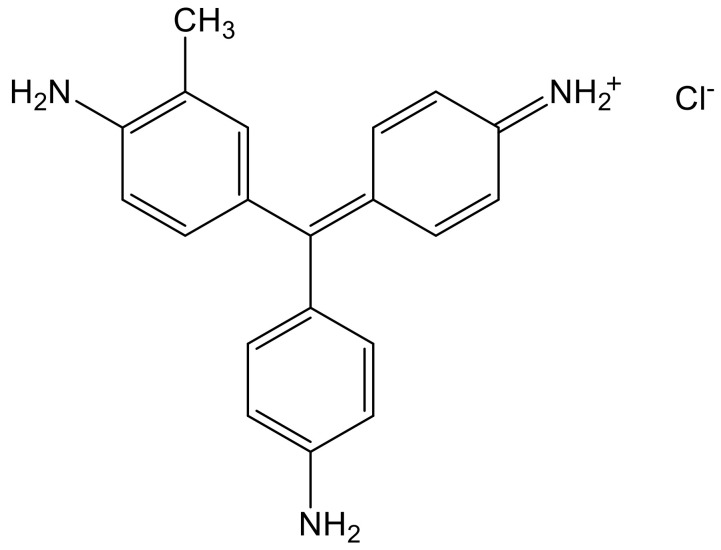
The structure of Fuchsine B.

Fuchsine B, monohydrochloride of 4-[(4-aminophenyl)(4-iminocyclohexa-2,5-dien -1-ylidene)methyl]-2-methylaniline, is a dye with magenta colour when dissolved in water and is used to stain bacteria due to its fluorescent properties. Fuchsine B (Figure 1) manifests acute toxicity, chronic aquatic toxicity, is a skin and eye irritant and is carcinogenic. 

In this respect, the discharge of fuchsine B from wastewaters represents a tremendous environmental importance. In the literature are reported several methods to remove fuchsine from aqueous solutions using peanut husk, which was crosslinked by epichlorohydrin [10], cheap nano-adsorbent derived from eggshell [11], carbons from lignin [12] or an ornamental epiphytic fern, originated from Hawaii and Africa, *Asplenium nidus* L. [13].

A large variety of adsorbents were successfully used in the last three years for various fuchsine dye derivatives removal from wastewater. Comparative adsorption capacities of fuchsine B in different stirring conditions, reported in recent literature and in this work, are presented in Table 1.

Metalloporphyrins with Pt and Zr, are only very recently proposed for discoloration/self-cleaning of waste waters, offering promising results [25,26]. Porphyrin-based porous organic polymer was also recently used [27].

Based on our team’s previous very good results, the purpose of this study was to extend the application of inorganic–inorganic or organic–inorganic hybrid silica matrices, as adsorbent materials, on fuchsine B contaminated wastewaters. 

In this context, modified silica matrices with platinum nanoparticles alone or in the presence of a porphyrin-base or with a Pt-metalloporphyrin originating from the same porphyrin-base, were comparatively investigated regarding their capacity to adsorb fuchsine B from wastewaters. The presence of platinum was required because of its known affinity toward amino groups.

## 2. Materials and Methods

### 2.1. Reagents

Hexachloroplatinic acid, trisodium citrate and fuchsine B (λmax = 543 nm) were provided by Sigma-Aldrich (St. Louis, MO, USA). Sodium borohydride was purchased from Merck (Darmstadt, Germany). Doubly distilled water was used in all investigations.

### 2.2. Synthesis of the Platinum Colloid

The platinum colloid was obtained according to previously published data [28] from H_2_PtCl_6_ × 6H_2_O twice reduced, firstly with trisodium citrate and secondly with NaBH_4._ Obtaining of platinum nanometric particles was accompanied by a change of color from yellowish to brownish yellow.

### 2.3. Catalyzed Sol-Gel Method in Acid/Base Two Steps for Obtaining Silica Hybrid Material

The procedure is similar to that already reported by our group [25]. A solution containing 20.84 g (0.1 mol) tetraethyl orthosilicate (TEOS) and 23.36 mL (18.44 g, 0.4 mol) ethilic alcohol (EtOH) was vigorusly stirred for 15 min and 0.165 mL (0.075 g, 0.002 mol) solution of 37% HCl was then added by slow dropping. The used molar ratios during the acid catalyzed step were: TEOS:EtOH:H_2_O:HCl = 1:4:6:0.02. The second base-catalyzed step was started after another 20 min, by slowly adding of 3 mL solution of 2.5% NH_3_. 

When an increase of the viscosity was observed, the matrix was divided in four and further impregnated with noble metal colloids and porphyrins, to obtain the same molar ratio of TEOS: impregnated material = 25,000:1 as follows: with 2.5 mL of 4 × 10^−6^ M Pt colloidal solution, to obtain ***Pt-silica material***; with 5 mL solution of 2 × 10^−6^ M PtTAOPP in tetrahydrofurane (THF), for ***PtTAOPP-silica material****;* with 5 mL solution of 2 × 10^−6^ M TAOPP in THF and 2.5 mL of 4 × 10^−6^ M Pt colloidal solution for ***(TAOPP-PtNPs)-silica material.***

The ***control silica material*** that was not impregnated showed as a transparent gel.

The wet gels were dried at 110 °C for 12 h and subsequently ground in mill.

### 2.4. Nitrogen Porosimetry

Textural characteristics of the samples were obtained by adsorption and desorption of N_2_ at 77 K, on a QuantachromeNova 1200 apparatus. The specific surface area (S_BET_) was obtained by the Brunauer–Emmett–Teller method.

### 2.5. Small Angle Neutron Scattering (SANS)

SANS measurements have been performed at the pin-hole type SANS instrument, called Yellow Submarine, located at Budapest Neutron Centre [29]. 

In a SANS measurement, the neutrons are scattered from the nanosized inhomogeneities of the sample, called scattering objects. The monochromatic neutrons scattered in angles smaller than 10 degrees are collected on a position-sensitive neutron detector. The collected neutron intensity is then radially averaged and plotted versus the scattering vector (Q), which is the defined as (1):

(1) $$Q=\frac{4\pi }{\lambda }\sin\theta$$

where λ is the wavelength of the neutron beam and θ is the half of the scattering angle.

The mathematical modelling of the SANS curves enables us to obtain qualitative and quantitative information about the scattering objects. Morphological and interfacial characteristics, orientation and dimension of the nanosized objects (having different scattering length density than their environment) are obtained from the total volume of the sample placed into the neutron beam.

The used wavelength range was 0.007–0.4 Å^−1^. The samples were placed in quartz cuvettes. The data were corrected with the standard correction procedure (background noise, detector pixel sensitivity, parameters of the instrument).

### 2.6. Scanning Electron Microscopy

The morphology and the surfaces of the samples were examined using scanning electron microscopy by a TESCAN 3 VEGA scanning electron microscope secondary electron detector (Brno, Czech Republic). All prepared samples were analyzed using 20 kV operating voltage and different magnifications up to 6000×.

### 2.7. Atomic Force Microscopy

The atomic force microscopy (AFM) images of the hybrid materials were registered on a Nanosurf^®^ EasyScan 2 Advanced Research AFM (Amsterdam, The Netherlands), equipped with a piezoelectric ceramic cantilever, in contact mode, after being drop-casted on silica pure plates. AFM data were quantitative on all three dimensions, the dark colours and their light tones representing the lower and the higher topography.

### 2.8. UV-VIS Spectroscopy

UV-visible spectra were registered on JASCO V-650 apparatus using 1 cm pathlength cells. Mill MM200 Retsch Mixer (Pfungstadt, Germany) equipped with grinding jars performing radial oscillations was used for obtaining silica powders. After the times established for the adsorption process, the supernatant solution was investigated by UV-vis spectroscopy to quantify the fuchsine B concentration that remains in liquid phase. The remaining concentration of fuchsine B was calculated in accordance with Lambert–Beer law, measuring the intensity of absorption at 544 nm and knowing the molar extinction coefficient. Each adsorption experiment was performed three times to ensure statistical requirements with insignificant deviations for average values.

### 2.9. Statistical Analysis 

Among the most complete statistical analyses for adsorption data that were reported in literature, hypothesis testing (using t test, paired t test and Chi-square test) was used to validate different conditions, such as: the optimum pH value for maximum removal; the efficiency of the experiment or for establishment of the relation between adsorbent quantity; and the removal efficiency of toxic substances [30,31]. The bootstrap method [32], studied the equilibrium concentrations in the fluid phase in relationship with the loadings of the solid; it was proved that by using this method the confidence ranges are wider than those predicted using traditional methods.

In order to ensure the reliability and reproducibility [33] in this work, the tests were performed in triplicate. The data were reported as the mean ± SD. 

## 3. Results and Discussion

### 3.1. Morphological Characterization

#### 3.1.1. Nitrogen Porosimetry

**Figure 2:**
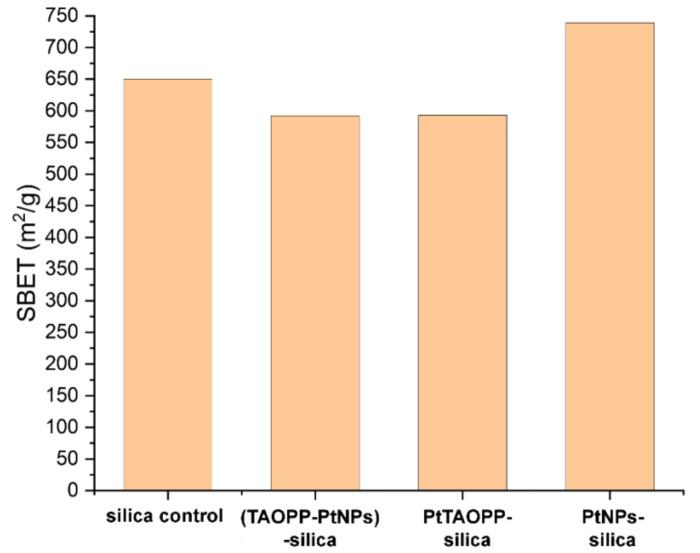
Specific surface area evolution for hybrid silica materials.

The specific surface area of the adsorbent materials has a deterministic effect on their performance. In order to perform textural characterization of the adsorbent nanomaterials nitrogen porosimetry was employed. All isotherms were type IVa, characteristic to the mesoporous materials [25,34]. The specific surface area evolution was presented in Figure 2. It is interesting to remark that the introduction of the porphyrins inside the silica support causes a 10% decrease of specific surface area while the introduction of the platinum nanoparticles causes a 14% increase. The specific surface areas were between 590–740 m^2^/g, proper for applications in adsorption processes. 

The specific surface areas were determined also after the fuchsine B adsorption and the results were very similar to those presented in Figure 2.

#### 3.1.2. Small Angle Neutron Scattering (SANS)

The nanostructure of the used materials for Fuchsine B adsorption was studied by the SANS method.

The scattered intensity versus scattering vector curves are shown in Figure S1 in the Supplementary File. The model fitting has been performed with the aid of the unified power-law and Guinier model, described by Equation (2).

(2) $$I\left(Q\right)=\mathrm{Aexp}\left(-\frac{Q^{2}{\mathrm{Rg}}^{2}}{3}\right)+B\frac{\mathrm{erf}{\left(\frac{\mathrm{QRg}}{\sqrt{6}}\right)}^{3p}}{Q^{p}}+\mathrm{Bg}$$

where A and B are scaling factors, Rg is the gyration radius, Bg is the incoherent scattering intensity.

The parameters of the SANS curve modeling are presented in Supplementary Files, Table S1.

The estimated diameter of the scattering objects has been calculated taking into account a spherical model, which is a broad approximation of the real shape. The scattering objects of the control sample are the silica nanoparticles forming the skeleton of the gel, with an average size of about 20 nm. 

The addition of the PtNPs to the control silica does not change the scattering curve profile, only the size of the particles is slightly decreased. The power exponent, characterizing the silica particles interface, is decreasing from 4 to 3.8 by the addition of the PtPNs. The exponent 4 is characteristic of smooth surfaces, while exponents between 3 and 4 are characteristic of fractal-like, rough surfaces. This is in a good agreement with the AFM results. 

By addition of the ***TAOPP***, the profile of the curve changes and the interface are characterized by a higher fractal dimension (D=6−p). When ***PtTAOPP*** is added to the silica, the fractal dimension increases even more, characterizing the surface interfaces with higher roughness. The presence of the ***TAOPP*** and ***PtTAOPP*** in the hybrid silica changed the nanostructure of the material, the particle size decreased to about the half, showing an average diameter of 10 nm. 

#### 3.1.3. SEM Characterization of the Hybrid Silica Materials after Fuchsine B Adsorption

**Figure 3:**
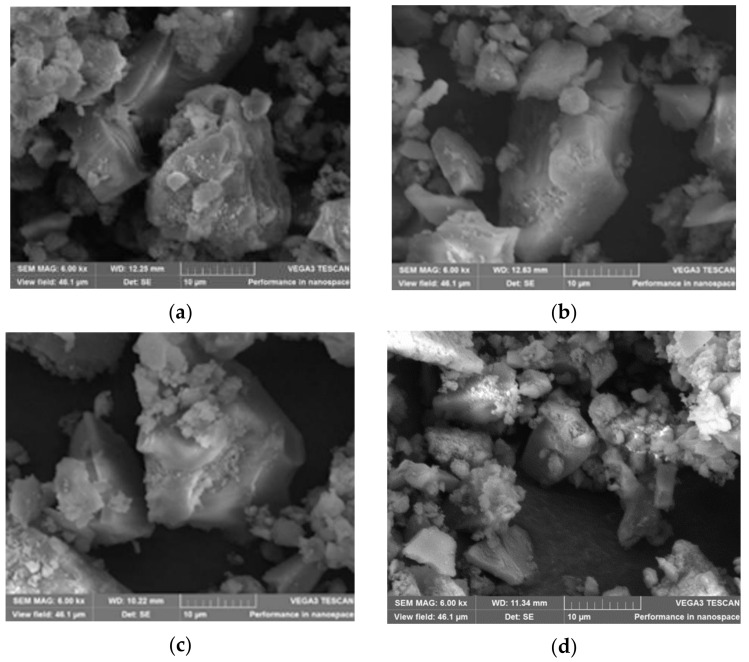
SEM images of the hybrid silica materials, at the same magnification, after complete adsorption of fuchsine B adsorption: (**a**) ***silica control***; (**b**) ***PtNPs-silica hybrid***; (**c**) ***(TAOPP-PtNPs)-silica hybrid***; (**d**) ***PtTAOPP-silica hybrid***.

Figure 3 presents the SEM images of the hybrid silica materials, at the same magnification, after complete adsorption of fuchsine B from solutions of 1 × 10^−6^ M solutions. No matter of the composition, the same characteristic stratified plate-like silica structures were visualized.

#### 3.1.4. AFM Investigation of the Hybrid Silica Materials after Fuchsine B Adsorption

**Figure 4:**
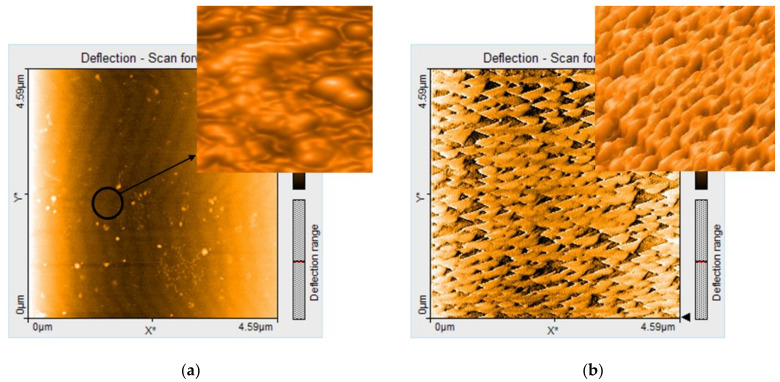

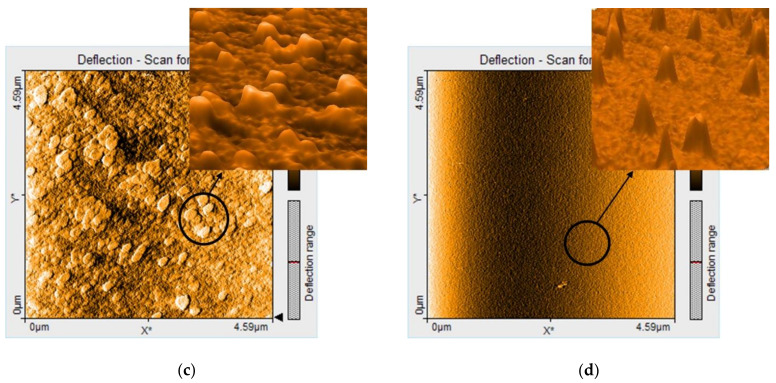
AFM images (4.59 µm × 4.59 µm view) of the silica hybrid materials after adsorption of fuchsine B: (**a**) **silica control**; (**b**) **PtNPs-silica hybrid**; (**c**) **(TAOPP-PtNPs)-silica hybrid**; (**d**) **PtTAOPP-silica hybrid.**

Figure 4 presents the 2D and 3D images obtained from AFM investigation, concerning the surface morphology and the architectures of the generated aggregates and/or self-assembles structures.

The microscopy analysis of ***PtNPs-silica hybrid*** after adsorption of fuchsine B shows uniform surfaces consisting of self-assembled isosceles building–block units with sides of around 220 nm, uniformly oriented, generating long wires, suggesting a significant J- type aggregation phenomenon, as can be seen in the 3D image. Large and uniformly distributed voids present on the surface of ***PtNPs-silica hybrid*** explain both physical adsorption but also chemisorption due to platinum affinity towards amino groups from fuchsine B. Besides, the largest specific surface area is a feature of ***PtNPs-silica hybrid.***

The morphology of the other silica materials is differently changing after fuchsine B adsorption, namely: ***(TAOPP-PtNPs)-silica hybrid*** and ***PtTAOPP-silica hybrid*** generated columnar type aggregates of different thickness, revealing a dominant H-type aggregation, columns that are randomly dispersed in the case of ***(TAOPP-PtNPs)-silica hybrid*** and uniformly distributed in the case of ***PtTAOPP-silica hybrid***. It seems that besides PtNPs, Pt-TAOPP metalloporphyrin also facilitates the chemisorption of fuchsine B molecules, due to interaction of the Pt atom coordinated in the center of the porphyrin macrocycle ring with the same amino functional groups.

The AFM images of silica control presents zigzags of multiple stacked ovoidal aggregates, changing the flow direction after a chevron patterning.

In all materials, the height distribution is ranging in the nanometric scale from 14 to 37 nm. The SANS and AFM results confirmed that the evolution of the surface roughness is maintained from nano to micro level and it is increasing in the following order: ***Silica control ˂*** ***PtNPs-silica hybrid ˂ (TAOPP-PtNPs)-silica hybrid ˂ PtTAOPP-silica hybrid***. In comparison with AFM images reported in [25], the materials after adsorption of fuchsine B are more structured, showing that the interaction between the fuchsine B and the silica-hybrid modified materials generated both H-type and J-type aggregations of different architectures, varying from triangular, to thin or thick columnar ones that finally self-oriented in parallel rows or after a chevron patterning.

### 3.2. Adsorption of Fuchsine B

#### 3.2.1. Testing of Impregnated-Silica Hybrid Materials and of Silica Control for Fuchsine B Removal from Wastewaters

Based on previously published results concerning the design of porphyrin silica hybrid materials with tailored pore sizes and shapes that are also exhibiting large specific surface areas [35,36], application of these novel hybrid materials for the removal of harmful/toxic dyes from solutions is promising, inexpensive and environmentally suited. 

#### 3.2.2. Method for Adsorption of Fuchsine B from Wastewaters, Monitored by Solid UV-Vis Spectra

Powders of identical quantities (0.01 g) from either the silica control or of each of the three modified-silica hybrid materials were introduced into test tubes, to obtain a loading of 3.3 g/L. Then 2.5 mL of 0.1 M NaOH solution containing 2.5 mL fuchsine B of different concentrations (in the range from 1 × 10^−4^ to 1 × 10^−7^ M) were added. The prepared mixtures were ultrasonically stirred for 60 s and then left for 3 h to observe the removal of colour. The pH = 13 of the solution favour the number of negatively charged hydroxyl groups from surface of the silica matrix, facilitating the attraction between the positively charged function of the fuchsine B and the adsorbent surface. 

**Figure 5:**
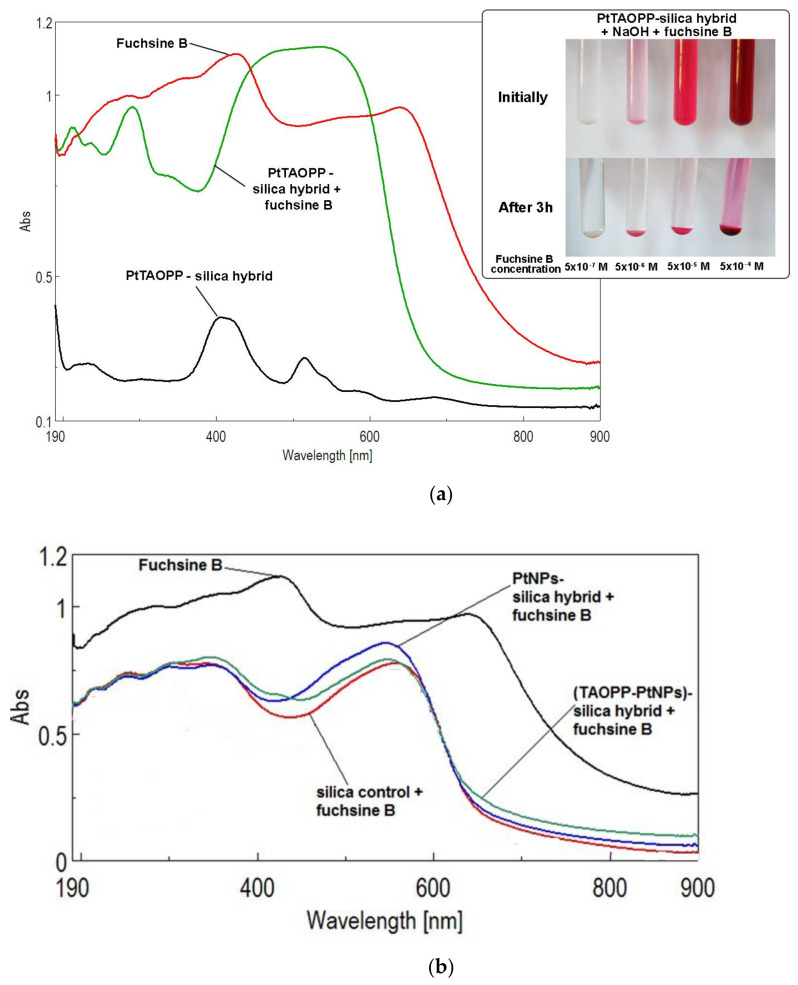
(**a**) The UV-Vis spectra recorded for solid ***PtTAOPP-silica hybrid*** before and after exposure to fuchsine B (with the following concentrations of fuchsine B: 1 × 10^−4^ M, 1 × 10^−5^ M, 1 × 10^−6^ M 1 × 10^−7^ M) as compared to the spectrum of solid fuchsine. A detail with the discoloration of the fuchsine B solution is displayed in the same image; (**b**) The UV-Vis spectra recorded for solid ***silica control, (TAOPP-PtNPs)-silica hybrid*** and ***PtNPs-silica hybrid*** after exposure to fuchsine B.

In Figure 5a it can be observed that after exposure to fuchsine the UV-vis spectrum of the hybrid material presents a widened and considerably more intense peak between 450 nm and 620 nm. This bathochromically shifted peak is the proof of the chemical interaction between the adsorbent material, ***PtTAOPP-silica hybrid*** and the fuchsine B dye. The inset presents the discoloration of the solutions. In Figure 5b a similar change of the shape of the UV-vis spectra can be noticed for each of the materials: ***silica control, (TAOPP-PtNPs)-silica hybrid*** and ***PtNPs-silica hybrid*** after exposure to fuchsine B, proving chemisorption phenomena.

#### 3.2.3. Adsorption of Fuchsine B from Wastewaters, Monitored by UV-Vis-Spectra in Water

**Figure 6:**
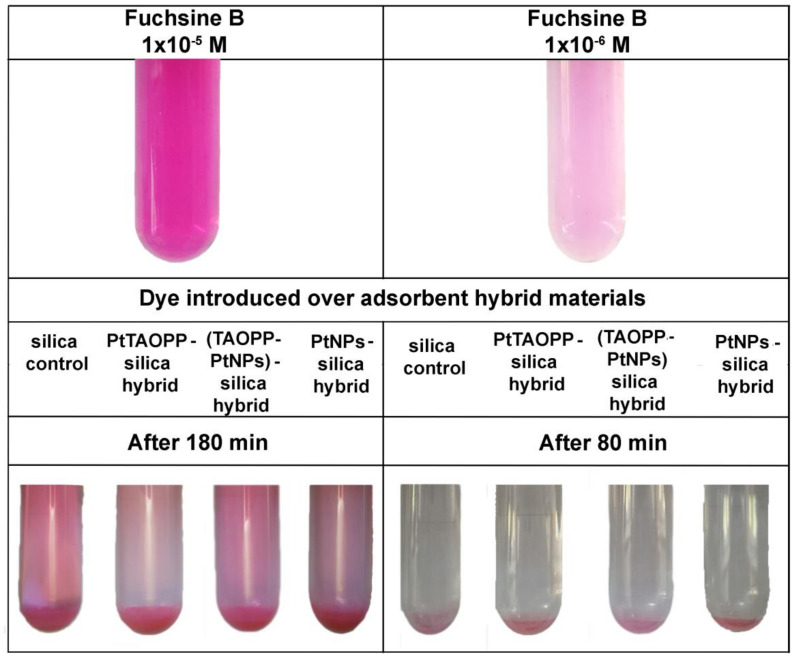
Photographs demonstrating the removal of color after a period of 180 and 80 min as a function of the Fuchsine B concentration (1 × 10^−5^ M and 1 × 10^−6^ M, respectively).

**Figure 7:**
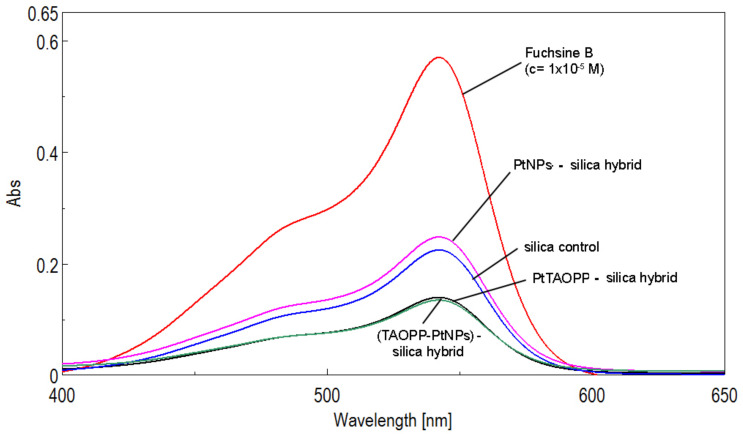
UV-vis spectra after 80 min of contact between the four adsorbent silica hybrid materials and the fuchsine B solution (initial concentration 1 × 10^−5^ M). The solutions were centrifuged and filtrated before UV-vis investigation.

After performing dye adsorption on silica control and on the other three modified-silica hybrid materials a significant discoloration of the fuchsine B solutions was noticed (Figure 6). This color removal was accompanied by a remarkable decrease in absorption intensity of the remaining fuchsine B in waste water-solution, represented in UV-vis spectra, as shown in Figure 7. 

Comparing the fuchsine B adsorption performances on silica control and on the other modified-silica hybrid materials, presented in Table 2 and in Figure 6 and Figure 7, it can be concluded that, regardless of the nature of silica adsorbent material, all the tested materials are capable of removing fuchsine from wastewaters. 

Nevertheless, the adsorption capacities of the silica materials containing porphyrins, namely ***PtTAOPP-silica hybrid*** and ***(TAOPP-PtNPs)-silica hybrid*,** are very similar and with 15–20% higher than in the case of silica control or silica impregnated only with platinum nanoparticles (Table 2). In addition, lower concentrations than 1 × 10^−5^ M fuchsine B are completely removed after 80 min, in still waters by all the proposed materials (Figure 6).

#### 3.2.4. Time Course Measurements

In order to establish the diminishing intensity of absorption of the fuchsine B function of contact time, the samples were prepared in quarz cuvettes by adding 3 mL solution at pH = 13 of fuchsine B (c = 1 × 10^−3^ M or c = 1 × 10^−4^ M in case of using silica control and ***PtTAOPP-silica hybrid***), over 0.01 g of each absorbent material (representing a loading of 3.33 g/L) and then stirring the mixture on vortex for 5 s. The cuvettes were then placed in the spectrophotometer taking care to start the measurements after 15 s from the contact of the fuchsine dye solution with the adsorbent, at the wavelength of 543 nm, for 1200 s.

**Figure 8:**
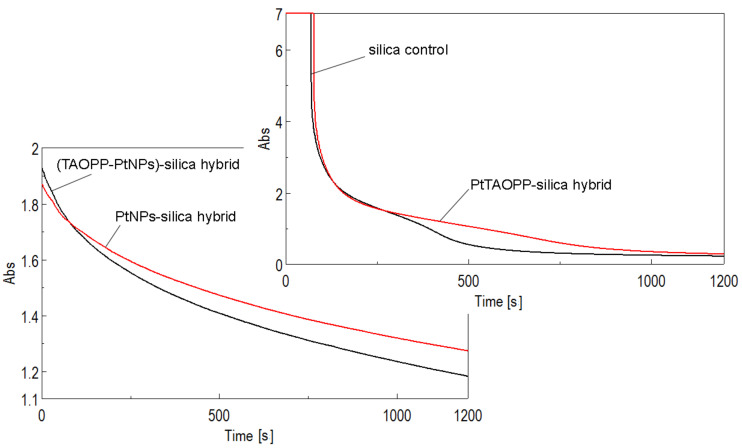
Time course measurements of the intensities of the samples containing fuchsine B (two different concentrations), in silica control and in modified silica hybrid adsorbent materials, at 543 nm.

Figure 8 presents the time course measurement for the case of fuchsine B.

The spectra presented in Figure 7 after approximately 150 s reveal a significant decrease in intensity of fuchsine B, meaning that a very fast adsorption process of fuchsine B is taking place on both ***PtTAOPP–silica material*** and on ***silica control***. After this period, the adsorption process slows down, although the graphs follow different courses. The final intensities of fuchsine B dye in the solutions have very low values, despite the fact that the initial absorbance intensities exceeded the reading capacity of the apparatus. Due to the fact that the final intensities are negligible, the conclusion is that both materials have the capacity to completely adsorb and remove fuchsine B dye from waste solution, at a concentration of 5 × 10^−4^ M, in a time interval of 1200 s, that means 58.67 mg fuchsine B/g adsorbent material, in good agreement with the best values mentioned in the literature: 58.8 mg/g in case of using raw pistachio nut shells [37], 58.48 mg/g in case of using HCl-modified malted sorghum mash [38] and 52.57 mg/g in the case of using epichlorohydrin crosslinked peanut husk [10]. 

#### 3.2.5. The Effect of Adsorbent Quantity upon the Adsorption of Fuchsine B, for Optimization of the Adsorption Process

The four silica materials: ***silica control***, ***PtTAOPP-silica hybrid***, ***(TAOPP-PtNPs)-silica hybrid*** and ***PtNPs-silica hybrid*** were further tested to determine the proper loading for achieving the best removal efficiency of fuchsine B from wastewaters.

The influence of the adsorbent quantity upon the removing of fuchsine was investigated for an initial dye concentration of 5 × 10^−4^ M at pH = 13, by using three different adsorbent loadings: 0.83 g/L, 1.66 g/L and 3.33 g/L, respectively, performing time course measurements at the maximum intensity of absorption of fuchsine B (wavelength of 543 nm), for 1200 s. 

**Figure 9:**
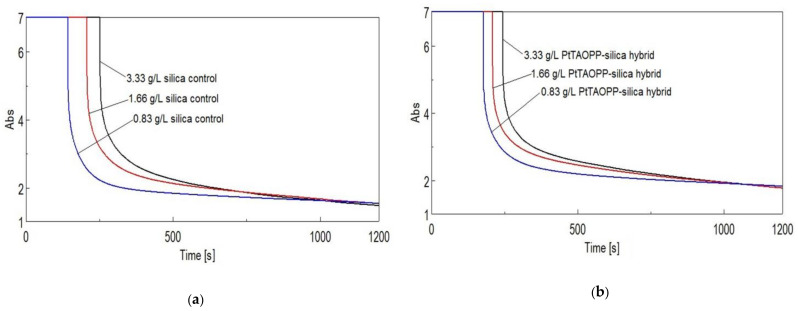

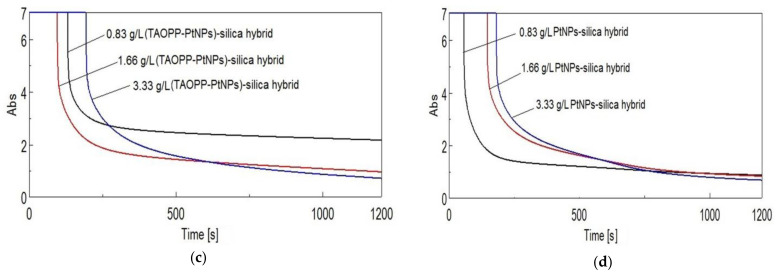
(**a**) The variation in time of the amount of fuchsine B dye adsorbed for the three different sorbent quantities of ***silica control***, (**b**) of ***PtTAOPP-silica hybrid***, (**c**) ***(TAOPP-PtNPs)-silica hybrid*** and (**d**) ***PtNPs-silica hybrid.***

Figure 9a–d shows the variation in time of the amount of fuchsine B dye adsorbed for three different sorbent quantities for each material: ***silica control*** (a), ***PtTAOPP-silica hybrid*** (b), ***(TAOPP-PtNPs)-silica hybrid*** (c) and ***PtNPs-silica hybrid*** (d). The best results for all four tested materials, ***silica control, PtTAOPP-silica hybrid (TAOPP-PtNPs)-silica hybrid*** and ***PtNPs-silica hybrid*** meaning the lower final absorption intensity of the fuchsine B, were obtained for the loading of 3.33 g/L. 

In case of using as adsorbents the ***silica control*** or ***PtTAOPP-silica hybrid***, the results are almost as good for the two lower loadings of 1.66 g/L and 0.83 g/L, meaning that these two materials are highly recommended for industrial use because of both financial and manufacturing benefits.

The amount of adsorbed fuchsine B was calculated with the Equation (3), according to previous published results [39]:

q_e_ = (C_i_ − C_e_)/S (mg/g) (3)

where: q_e_ represents the amount of adsorbed dye (mg/g); C_i_ represents the initial concentration of dye in solution (mg/L); C_e_ represents the equilibrium or final dye concentration (mg/L) and S represents the dosage concentration, mass of sorbent/volume unit (g/L). The percentage of dye removal is calculated according to the Equation (4). Both results are presented in Table 3.

Percent of dye removal = (C_i_ − C_e_)/C_i_ × 100 (4)

The best adsorption capacity of fuchsine B, which is 197.28 mg/g of adsorbent, is offered by ***PtNPs-silica hybrid****,* followed by silica control which is in full agreement with the specific surface area sizes (Figure 2).

Further kinetic studies were performed on the ***PtNPs-silica hybrid*** and ***PtTAOPP-silica hybrid*** to establish the mechanism of fuchsine B dye adsorption and were presented in Supplementary files, as follows: the influence of silica materials loading upon the adsorption of Fuchsine B, in Figure S2. The effect of varying of the initial fuchsine B concentration and of contact time, in Figure S3; influence of initial fuchsine B concentration on the adsorption capacities of ***PtNPs-silica hybrid*** and ***PtTAOPP-silica hybrid*** for 0.83 g/L adsorbent loading, in Table S2; Lagergren plots for ***PtNPs-silica hybrid*** and ***PtTAOPP-silica hybrid*** at different loadings for the initial concentration of fuchsine B of 5 × 10^−4^ M, shown in Figure S4; and kinetic parameters for the adsorption of fuchsine B at different loadings, presented in Table S3. In conclusion, it was observed that the second order kinetic model fits better with the experimental data, confirming that the adsorption takes place involving both physical and chemical processes, a similar result with that reported in [25] for methylene blue.

### 3.3. Desorption Studies

**Figure 10:**
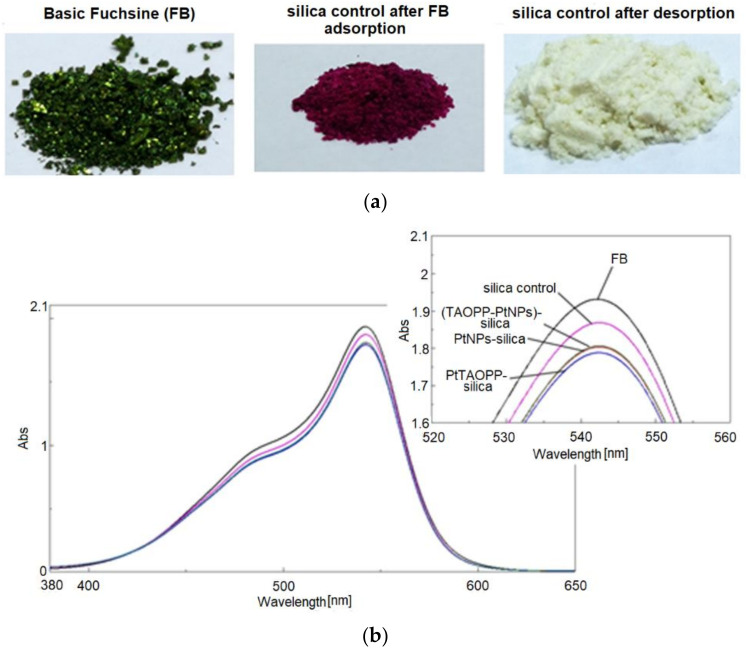
(**a**) The aspect of solid dye and of silica control after adsorption and after desorption tests, (**b**) UV-vis of the supernatant solutions obtained after desorption with NaOH from different adsorbents in comparison with the initial UV-vis spectrum at Ci concentration of Fuchsine B.

The desorption process was conducted by the addition of 5 mL NaOH 0.5 N, under stirring and heating to 60 °C, to each filtered silica material that adsorbed Fuchsine B using the mass of sorbent 3.33 g/L. The immediate discoloration of the adsorbent accompanied by the intensification of the color of the supernatant was observed. The aspect of solid dye and of silica control after adsorption and after desorption tests are presented in Figure 10a, together with the UV-vis of the supernatant solutions obtained after desorption with NaOH from different adsorbents, within 2 h, (Figure 10b) in comparison with the initial concentration C_i_ of Fuchsine B. As can be easily seen from Figure 10a,b, silica control is the material that can be completely regenerated and releases almost all the adsorbed dye. The materials so recovered (after being twice washed with distilled water and dried in the oven), can be re-used at least three times. 

## 4. Conclusions

The main goal of this work was to extend the application of efficient silica materials capable to remove methylene blue, malachite green and Congo red from waste waters, to fuchsine B.

The morphology of the used silica and modified-silica materials has been studied. Typical stratified plate-like silica particles were obtained no matter of the silica hybrid composition. AFM and SANS confirmed the modification of the surface roughness of the hybrid materials by using porphyrins or platinum nanoparticles. The surface roughness is maintained from nano to micro level and it is increasing in the following order: ***silica control* ˂ *PtNPs-silica hybrid* ˂ *(TAOPP-PtNPs)-silica hybrid* ˂ *PtTAOPP-silica hybrid***.

Fuchsine B removal from waste waters were performed on silica control and on modified-silica hybrids and showed adsorption capacities around 197.28 mg/g for ***PtNPs-silica hybrid***, very similar to the best ones reported in the literature [19,24] and three times higher than the performances obtained for methylene blue [25]. The ***PtNPs-silica material*** increased adsorption capacities have been explained by the large voids in the aggregated architectures with capacity of self-organization. The physical adsorption might be accompanied by chemisorption due to platinum affinity towards amino groups from fuchsine B. In addition, the largest specific surface area, that is a feature of the ***PtNPs-silica hybrid***, might offer more active sites for interaction with the dye.

The adsorption depends on fuchsine B concentrations, the values of the specific surface area of each material and the dosage concentration of the silica-hybrid adsorbent materials. Kinetic studies reveal that the second order kinetic model fits better with the experimental data, confirming that the adsorption takes place involving both physical and chemical processes. Chemisorption of fuchsine B is favoured in comparison with methylene blue, on the same adsorbent materials, because Fuchsine B contains two primary amine groups, able to interact with hydrogen at the surface of silica hybrids.

The most important result is that the absorption process of fuchsine B removal is of 100% performance in very coloured solutions of 10^−5^ M concentration, being a fast process, expanding in this way the environmental applicability. Yields in the range of 86–98% were obtained by all tested materials for adsorption of fuchsine B in a higher concentration range, from 10^−3^ to 5 × 10^−4^ M. Besides, by a simple procedure, the materials can be recovered and reused at least 3 times.

## Figures and Tables

**Table 1:** Comparative adsorption capacities of fuchsine B in different stirring conditions, reported in recent literature.

Adsorbent for Fuchsine Dyes	Adsorbtion Capacity (mg/g)	Surface Area (m^2^/g)	Reference
Calcined Mussel Shell	141.65	-	[14]
Magnetized bio carbon from peanut shell	19.64 In acid form	-	[15]
Porous Cu_2_FeSnS particles	123.12 In acid form	53.5	[16]
(Acrylamide-*co*-Sodium methacrylate)-graft-Chitosan gel	6.07	spongeous surface with pores of around 10 μm	[17]
Powdered biosorbent from the mandacaru cactus (*cereus jamacaru*) leaves	324.5	surface with cavities and protrusions	[18]
Zeolite made from sodium metasilicate as silicon precursor and impregnated with Fe(III) cations using tetrapropylammonium bromide	205.83	399	[19]
Mesoporous Si−Al material, prepared by alkali calcination leaching of natural palygorskite, and sequent hydrothermal synthesis coupled with calcination	54.4	995	[20]
Hard shell of *Euryale ferox* seeds	19.48	-	[21]
Mesoporous silica materials prepared via co-condensation of silicon from natural mineral palygorskite by alkali-melting extraction (SBA-16)	39.61	687.23	[22]
SBA-16 functionalized with aluminium	70.08	514.39	[22]
amorphous eggshell membrane porous powder	47.85	11.56	[23]
Starch-capped zinc selenide nanoparticles loaded on activated carbon	222.7	-	[24]
Silica hybrid materials impregnated with platinum nanoparticles (PtNPs)	197.28	739 ± 19	This work
Silica hybrid materials impregnated with Pt-metalloporphyrin (PtTAOPP)	190.46±	593±15	This work

**Table 2:** Fuchsine B adsorption capacity of the tested silica materials.

Adsorbent Material	Fuchsine B Concentration 1 × 10^−5^ M		Fuchsine B Concentration 1 × 10^−6^ M	
	q_e_ (mg\g)	η (%)	q_e_ (mg\g)	η (%)
Silica control	6.2 ± 0.01	60.7	1.02 ± 0.002	100
PtTAOPP-silica hybrid	7.9 ± 0.01	76.6		
(TAOPP-PtNPs)-silica hybrid	7.7 ± 0.01	75.7		
PtNPs-silica hybrid	5.7 ± 0.01	56.63		

**Table 3:** Influence of the adsorbent loading of ***silica control***, ***PtTAOPP-silica hybrid***, (***TAOPP-PtNPs)-silica hybrid*** and ***PtNPs-silica*** ***hybrid*** on the adsorption capacity of fuchsine B.

Adsorbent Material	Mass of Sorbent (g/L)					
	0.83		1.66		3.33	
	q_20min_ (mg/g)	η (%)	q_20min_ (mg/g)	η (%)	q_20min_ (mg/g)	η (%)
***Silica control***	192.55 ± 0.2	94.62	96.29 ± 0.1	94.64	48.10 ± 0.06	94.54
***PtTAOPP-silica hybrid***	190.46 ± 0.15	93.59	95.43 ± 0.1	93.79	47.57 ± 0.06	93.79
***(TAOPP-PtNPs)-silica hybrid***	168.10 ± 0.1	86.43	98.38 ± 0.1	96.69	49.47 ± 0.05	98.91
***PtNPs-silica hybrid***	197.28 ± 0.1	96.94	98.81 ± 0.1	97.11	49.52 ± 0.05	98.96

## Data Availability

All the data are available at the Corresponding author and offered if requested.

## Supplementary Materials

The following are available online at https://www.mdpi.com/article/10.3390/nano11040863/s1, Figure S1: Scattered neutron intensities versus the scattering vector (dots) and the mathematical model fitting (continuous line) of the curves, Figure S2: The variation in time of the amount of dye adsorbed by the three adsorbent loadings investigated (**a**) ***PtNPs-silica hybrid*** (**b**) ***PtTAOPP-silica hybrid***, Figure S3: The influence of the initial concentration of dye and contact time onto the Fuchsine B removal (pH = 13), 298 K, 0.83 g/L adsorbent loading for (**a**) ***PtNPs-silica hybrid*** and (**b**) ***PtTAOPP-silica hybrid***, Figure S4: Lagergren plots for ***PtNPs-silica hybrid*** and ***PtTAOPP-silica hybrid*** at different loadings for the initial concentration of Fuchsine B of 5 × 10^−4^ M, Table S1: Parameters of the SANS curve modeling, Table S2: Influence of initial FB concentration on the adsorption capacities of ***PtNPs-silica hybrid*** and ***PtTAOPP-silica hybrid*** for 0.83 g/L adsorbent loading, Table S3: Kinetic parameters for the adsorption of Fuchsine B by ***PtNPs-silica hybrid*** and ***PtTAOPP-silica hybrid*** at different loadings
