# Supplementary material for: Hybrid Silica Materials Applied for Fuchsine B Color Removal from Wastewaters
Source: Nanomaterials (Basel). 2021 Mar 28;11(4):863. doi: 10.3390/nano11040863 (PMC8066414; doi:10.3390/nano11040863)
Supplement: Supplementary file 1 [file nanomaterials-11-00863-s001.pdf]

Supplementary Material

Hybrid Silica Materials Applied for Fuchsine B Color Removal from Wastewaters

Ion Fratilescu [1], Zoltán Dudás [2], Mihaela Birdeanu [3], Camelia Epuran [1], Diana Anghel [1], Ionela Fringu [1], Anca Lascu [1], Adél Len [2,4] and Eugenia Fagadar-Cosma [1,*]

[1] Institute of Chemistry "Coriolan Dragulescu", Mihai Viteazu Ave. 24, 300223 Timisoara, Romania; ionfratilescu@acad-icht.tm.edu.ro (I.F.); ecamelia@acad-icht.tm.edu.ro (C.E.); danghel@acad-icht.tm.edu.ro (D.A.); mcreanga@acad-icht.tm.edu.ro (I.F.); alascu@acad-icht.tm.edu.ro (A.L.)
[2] Centre for Energy Research, Neutron Spectroscopy Department,, Konkoly-Thege Street 29-33, 1121 Budapest, Hungary; dudas.zoltan@ek-cer.hu (Z.D.); len.adel@ek-cer.hu (A.L.)
[3] National Institute for Research and Development in Electrochemistry and Condensed Matter, P. Andronescu Street 1, 300224 Timisoara, Romania; mihaione2002@yahoo.com
[4] Civil Engineering Department, University of Pécs, Boszorkány Street 2, 7624 Pécs, Hungary
* Correspondence: efagadar@yahoo.com or efagadarcosma@acad-icht.tm.edu.ro

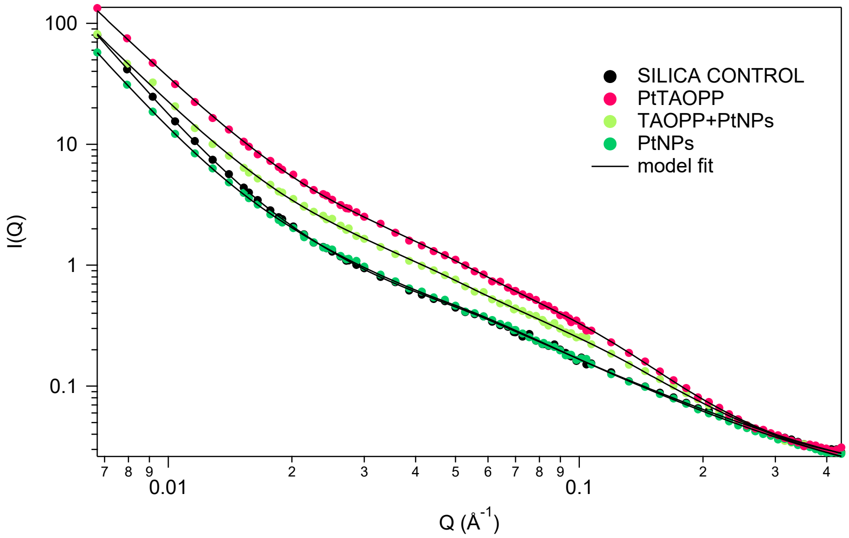

Figure S1. Scattered neutron intensities versus the scattering vector (dots) and the mathematical model fitting (continuous line) of the curves.

Table S1. Parameters of the SANS curve modeling.

Sample	R_g (Å)	Approximated diameter (nm)	p
Silica control	78	20.28	4
PtNPs - silica	77	20.02	3.8
TAPP - PtNPs - silica	40	10.4	3.4
PtTAOPP - silica	36	9.36	3.3

Adsorbtion Studies

The Effect of Silica Adsorbent Materials Loading upon the Adsorption of Fuchsine B

Three different silica adsorbent materials loadings: 0.83 g/L, 1.66 g/L and 3.33 g/L were used to investigate the influence of the adsorbent weight quantity upon the adsorption of FB having a fixed initial concentration of 5 x 10^{-4} M (168.9 mg/L). In each case, time course measurements of the intensity of absorption of Fuchsine B were performed at the wavelength of 542 nm, for 1200 seconds. Figure S2 (a and b) shows the variation in time of the amount of FB dye adsorbed for the three adsorbent loadings investigated, for (a) ***PtNPs-silica hybrid*** and (b) for ***PtTAOPP-silica hybrid***.

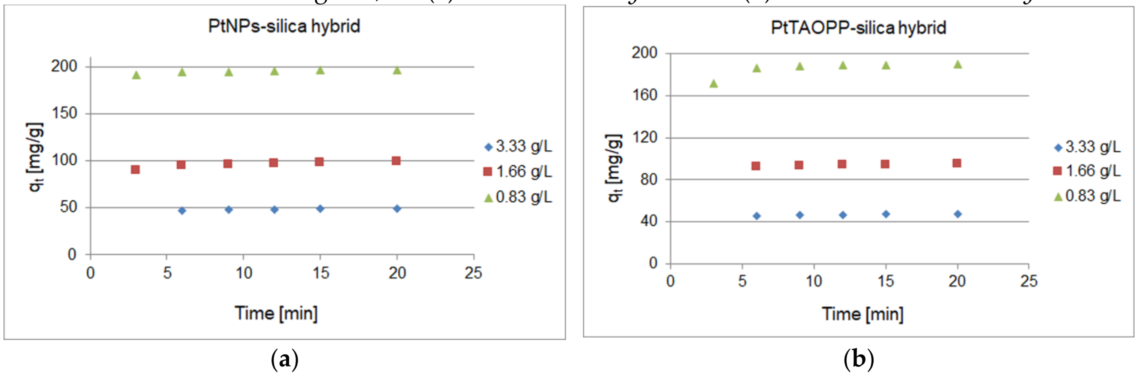

Figure S2. The variation in time of the amount of dye adsorbed by the three adsorbent loadings investigated (**a**) ***PtNPs-silica hybrid*** (**b**) ***PtTAOPP-silica hybrid.***

The Effect of Varying of the Initial FB Concentration and Contact Time

The plots between the amount of adsorbed dye as a function of time, when using different FB concentrations (8.445 mg/L; 16.89 mg/L; 84.4 mg/L; 168.9 mg/L) are presented in Figure S3. From Figure S3 a remarkable and continuous adsorbtion of FB at its highest concentration of 168.9 mg/L can be noticed in case of using ***PtNPs-silica hybrid*** as adsorbent material, but ***PtTAOPP-silica hybrid*** shows also a very good result.

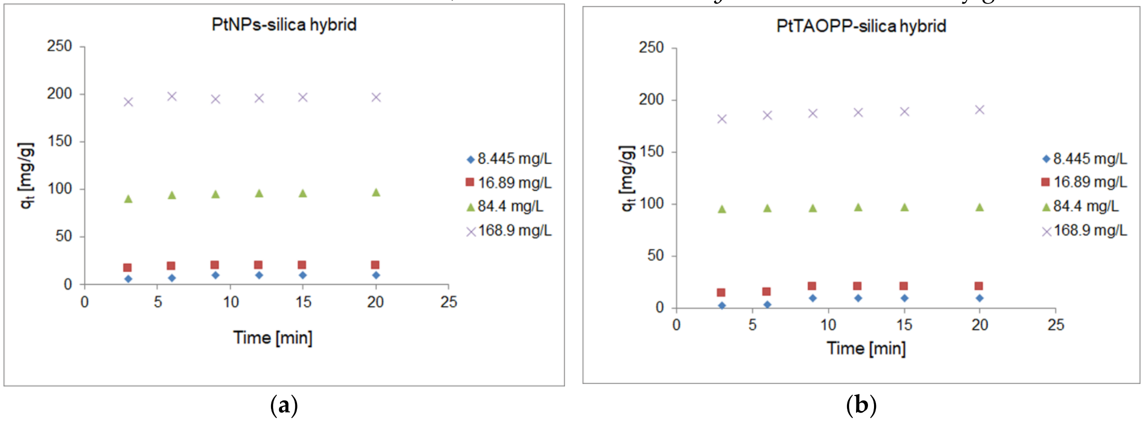

Figure S3. The influence of the initial concentration of dye and contact time onto the Fuchsine B removal (pH = 13), 298 K, 0.83 g/L adsorbent loading for (**a**) ***PtNPs-silica hybrid*** and (**b**) ***PtTAOPP-silica hybrid.***

Table S2. Influence of initial FB concentration on the adsorption capacities of ***PtNPs-silica hybrid*** and ***PtTAOPP-silica hybrid*** for 0.83 g/L adsorbent loading

Adsorbent	Initial FB concentration [mg/L]	Adsorption capacity $q_{20\ min}$ [mg/g]	Yield η [%]
PtNPs-silica hybrid	8.445	10.17 ± 0.5	100
PtTAOPP-silica hybrid		10.17 .± 0.4	100
PtNPs-silica hybrid	16.89	20.35 ± 0.1	100
PtTAOPP-silica hybrid		16.941 ± 0.3	100
PtNPs-silica hybrid	84.4	97.174 ± 0.4	95.56
PtTAOPP-silica hybrid		97.598 ± 0.2	95.92
PtNPs-silica hybrid	168.9	197.28 ± 0.1	96.94
PtTAOPP-silica hybrid		190.46 ± 0.5	93.59

Kinetic Studies for the Adsorption of Fuchsine B on *PtNPs-silica hybrid* and *PtTAOPP-silica hybrid*

First order kinetic study:

Kinetic studies provide information concerning the mechanism of dye adsorption. According to [1] the pseudo-first-order kinetic of adsorption is represented by Equation (S1):

$$\frac{dq_t}{dt} = k_t(q_e - q_t) \qquad (S1)$$

where q_t is the amount of dye adsorbed at time t (mg/g); q_e is the adsorption capacity at equilibrium (mg/g); k_t is the pseudo first order rate constant (min^{-1}); t is the contact time (min). The integration of this equation with initial conditions ($q_t = 0$ at $t = 0$) leads to the following Equation (S2):

$$\mathbf{\log(q_e - q_t) = \log q_e - \frac{k_1}{2.303}t} \qquad (S2)$$

The value for k_1 rate constant is calculated from the linear plots of $\log(q_e\text{-}q_t)$ versus t**.** Lagergren Plots, as the Slope of the Plots [2].

The parameters for a first order kinetic process can be calculated from Lagergren plots for ***PtNPs-silica hybrid*** and ***PtTAOPP-silica hybrid*** at different loadings for the initial concentration of Fuchsine B of 5×10^{-4} M, that are shown in Figure S4.

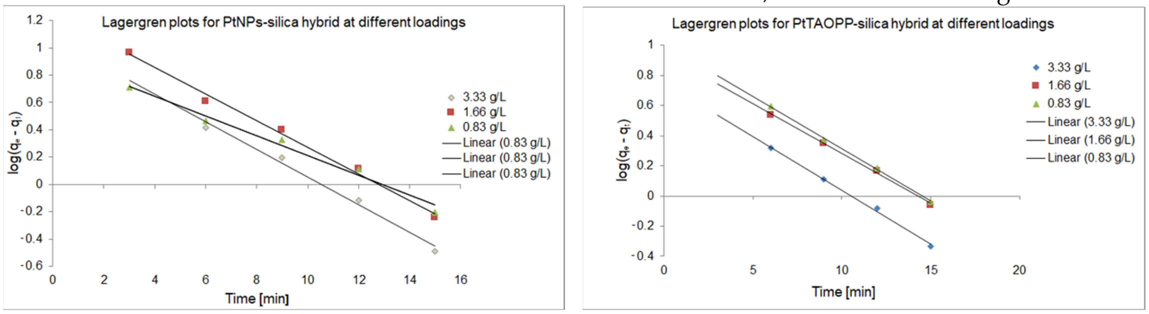

(**a**) (**b**)

Figure S4. Lagergren plots for ***PtNPs-silica hybrid*** and ***PtTAOPP-silica hybrid*** at different loadings for the initial concentration of Fuchsine B of 5×10^{-4} M.

Because the Lagergren plots are linear for the investigated loading of adsorbent materials, the kinetics of the adsorption process could be attributed as a first order kinetic

mechanism. Nevertheless, the calculated values for q_t (Table S3) are not in accordance with the experimental ones.

Second Order Kinetic Study

Next we calculated the second order kinetic parameters presented in the Table S3 for a FB concentration of 5×10^{-4} M. The pseudo-second order adsorption kinetic model was applied for both adsorbent materials and the rate constant of pseudo-second order adsorption was calculated from Equation (S3), representing the integrated pseudo-second order adsorption kinetic rate to initial conditions qt = 0 at t = 0 [2]:

$$\frac{1}{q_e - q_t} = \frac{1}{q_e} + k_2 t \quad \text{(S3)}$$

where k_2 is the rate constant of pseudo-second order adsorption (g min^{-1} mg^{-1}).

The initial adsorption rate constant, *h* (mg g^{-1} min^{-1}) as $q_t/t \rightarrow 0$ at t = 0 can be defined as Equation (S4) [2]:

$$h = k_2 q_e^2 \quad \text{(S4)}$$

Table S3. Kinetic parameters for the adsorption of Fuchsine B by ***PtNPs-silica hybrid*** and ***PtTAOPP-silica hybrid*** at different loadings**.**

Equations	Parameters	***PtNPs-silica hybrid***			***PtTAOPP-silica hybrid***		
		**0.83 g/L**	**1.66 g/L**	**3.33 g/L**	**0.83 g/L**	**1.66 g/L**	**3.33 g/L**
**Pseudo-first order**	q_e exp. [mg/g]	197.28	98.81	49.52	190.47	95.43	47.57
	q_e calc. [mg/g]	518.738	201.912	96.347	362.173	212.338	95.099
	k_1[min^{-1}]	0.0718	0.0963	0.0741	0.108	0.062	0.0716
**Pseudo-second order**	q_e calc. [mg/g]	197.03	97.75	40.95	188.198	95.269	48.553
	k_2[g mg^{-1} min^{-1}]	0.068	0.059	0.109	0.046	0.0567	0.1
	h [mg g^{-1} min^{-1}]	2646.47	576.05	269.5	1668.82	516.36	226.29

It can be observed that the second order kinetic model fits better with the experimental data, confirming that the adsorption takes place implying both physical and chemical processes.

References

1. Kaur, S.; Rani, S.; Mahajan, R. K. Adsorption Kinetics for the Removal of Hazardous Dye Congo Red by Biowaste Materials as Adsorbents. *J. Chem* **2012**, *2013*, 1–12, DOI:/10.1155/2013/628582
2. Lyubchik, S.; Lygina, E.; Lyubchyk, A.; Lyubchyk, S.; Loureiro J.M.; Fonseca I.M.; Ribeiro A.B.; Pinto, M.M.; Sa Figueiredo A.M. *The Kinetic Parameters Evaluation for the Adsorption Processes at "Liquid–Solid" Interface. In: Electrokinetics Across Disciplines and Continents New Strategies for Sustainable Development*, Ribeiro, Alexandra B., Mateus, Eduardo P., Couto, Nazaré, Eds. Springer International Publishing Cham Switzerland 2016. ISBN 978-3-319-20179-5. 2016, pp. 81–109. DOI: 10.1007/978-3-319-20179-5_5
